# Pyrolytic Waste Plastic Oil and Its Diesel Blend: Fuel Characterization

**DOI:** 10.1155/2016/7869080

**Published:** 2016-06-28

**Authors:** M. Z. H. Khan, M. Sultana, M. R. Al-Mamun, M. R. Hasan

**Affiliations:** Department of Chemical Engineering, Jessore Science and Technology University, Jessore 7408, Bangladesh

## Abstract

The authors introduced waste plastic pyrolysis oil (WPPO) as an alternative fuel characterized in detail and compared with conventional diesel. High density polyethylene, HDPE, was pyrolyzed in a self-designed stainless steel laboratory reactor to produce useful fuel products. HDPE waste was completely pyrolyzed at 330–490°C for 2-3 hours to obtain solid residue, liquid fuel oil, and flammable gaseous hydrocarbon products. Comparison of the fuel properties to the petrodiesel fuel standards ASTM D 975 and EN 590 revealed that the synthetic product was within all specifications. Notably, the fuel properties included a kinematic viscosity (40°C) of 1.98 cSt, density of 0.75 gm/cc, sulphur content of 0.25 (wt%), and carbon residue of 0.5 (wt%), and high calorific value represented significant enhancements over those of conventional petroleum diesel fuel.

## 1. Introduction

Plastics have become an indispensable part in today's world, due to their lightweight, durability, and energy efficiency, coupled with a faster rate of production and design flexibility; these plastics are employed in entire gamut of industrial and domestic areas; hence, plastics have become essential materials and their applications in the industrial field are continually increasing. At the same time, waste plastics have created a very serious environmental challenge because of their huge quantities and their disposal problems. Waste plastic pyrolysis in liquid fuel (gasoline, diesel oil, etc.) or chemical raw materials not only can effectively solve the problem of white pollution, but also can alleviate the energy shortage to a certain extent. Recycling of waste plastics is expected to become the most effective way. Waste plastics' recycling, regenerating, and utilizing have become a hot spot of research at home and abroad and gradually formed a new industry [1–6].

The decomposition of polymeric materials is also relevant and of interest to industries since plastic is used in many of today's commodities [7, 8]. The wide use of polymeric materials or plastics resulted in the accumulations of untraditional wastes not native to the mother earth life cycle [9, 10]. Therefore, wastes of modern materials are accumulated without effective decomposition and recycling routes in the landfills. The increase of petroleum and petrochemical prices opened the ways for industries to invest in decomposition of plastic wastes to petrochemicals [11, 12]. Today, plastic landfills are as valuable as petroleum mines. Models for reaction's kinetics for optimal pyrolysis conditions of plastic waste mixtures have been proposed by researchers. Literature abounds in the recycling of these traditional wastes to petrochemicals [13–15] and many industries are sustained and developed based on decomposition of natural and synthetic polymers [14, 15]. From a scientific-engineering point of view, nondegradability of plastics is no longer an environmental issue in landfills since the plastics can be recycled. However, run-away plastic wastes are continuing to be a huge hazard on the surface and surface water such as waterways, seas, and oceans, endangering safe life for both animals and humans [15].

The plastics include polystyrene [16, 17], poly (vinyl chloride) [17, 18], polypropylene [17–19], PE terephthalate [18], acrylonitrile-butadiene-styrene [18], and PE [16–18]. In some cases, plastics were copyrolyzed with other materials such as waste motor oil [18]. With regard to fast pyrolysis of PE, pyrolysis of LDPE [16], HDPE [20, 21], and various mixtures [17] was reported. In all PE studies, the properties of the resulting bio-oil were not reported, nor were the upgrading to fuel-grade hydrocarbons and subsequent fuel property determination.

The objective of this study was the production, characterization, and evaluation of alternative diesel fuel from pyrolysis of HDPE waste plastics. Comparison of our pyrolyzed oil with conventional petroleum-derived diesel fuel was a further objective, along with a comparison to petrodiesel standards such as ASTM D 975 and EN 590. Blends of waste plastic pyrolysis oil (WPPO) with diesel were prepared and the resultant fuel properties were measured. It is anticipated that these results will further the understanding of the applicability and limitations of HDPE as a feedstock for the production of alternative diesel fuel.

## 2. Materials and Methods

### 2.1. Materials and Process Description

The plastic used in this study was used waste plastic containers (HDPE) for domestic purposes. Waste plastics were cleaned with detergent and water to remove contained foreign materials such as mud and oil. Washed out waste plastics were dried and cut into small pieces in the range of 0.5 inches to 2 inches by using scissor.

### 2.2. Experimental Setup

**Figure 1:**
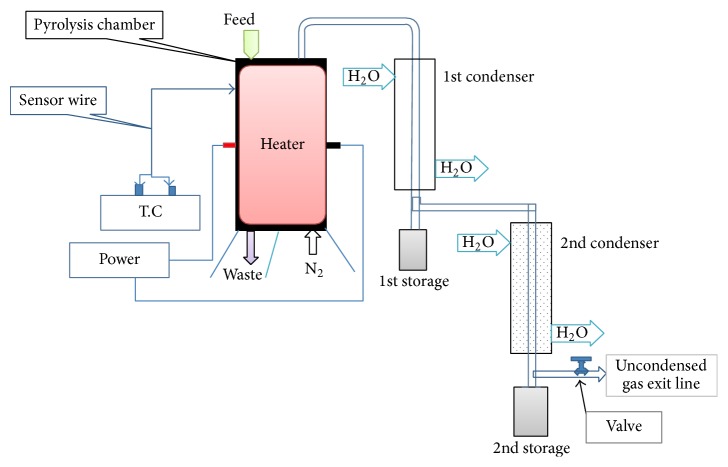
Schematic representation of experimental setup.

A laboratory scale externally heated fixed bed pyrolysis batch reactor was used for production of oil from plastic.  Figure 1 shows the schematic diagram of plastic pyrolysis setup. Basic instruments of the pyrolysis chamber are temperature controller, condenser, temperature sensor, a heating coil, insulator, storage tank, valve, and gas exit line. The effective length and diameter of the stainless steel made reactor are 38 cm and 15 cm, respectively. The reactor with tire was heated electrically up to 475°C with Ni-Cr wire electric heater. Here it is necessary to mention that the sensor was used through the wall of the stainless steel pyrolysis chamber to measure the temperature. Therefore, the temperature mention may have appeared small in amount as compared to conventional system. Besides, a nitrogen hole was used in the pyrolysis chamber to provide uniform heating across the cross-section of the reactor chamber and to create inert environment in the pyrolysis chamber.

There was no output at low temperature range and the process was carried out between the temperature ranges of 330°C and 490°C in the reactor for about two hours and forty minutes. The vapor products of pyrolysis were carried out through two condensers. The condensers were cooled by water and the condensed bio-oil was collected into two collectors. The noncondensed gas was flared to the atmosphere and the char was collected from the reactor after completion of pyrolysis cycle.

### 2.3. Fuel Properties

All the fuel properties of the oil were tested by the following methods which are summarized in Table 1.

The density measurement is done with accuracy of ±0.0005 g/mL and the other parameters such as pour point, flash point, and fire point are measured with ±1°C accuracy.

## 3. Results and Discussion

### 3.1. Effect of Temperature on Product Yield

**Figure 2:**
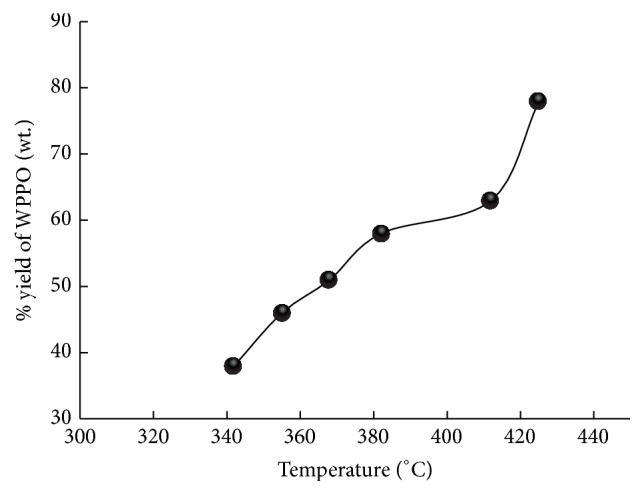
Effect of temperature on product yield.

The products are separated into gas, oil, and char residue by pyrolysis of waste plastic. About 38.5% of WPPO was obtained at temperature 330°C as presented in Figure 2. The oil percentage increased constantly to 76.0% at 425°C. The gases produced through plastic pyrolysis consist principally of hydrogen (H_2_), carbon dioxide (CO_2_), carbon monoxide (CO), methane (CH_4_), ethane (C_2_H_4_), and butadiene (C_4_H_6_), with trace amounts of propane (CH_3_CH_2_CH_3_), propene (CH_3_CH=CH_2_), n-butane (CH_3_(CH_2_)_2_CH_3_), and other miscellaneous hydrocarbons.

#### 3.1.1. Effect of Distillation Temperature on Crude WPPO

**Figure 3:**
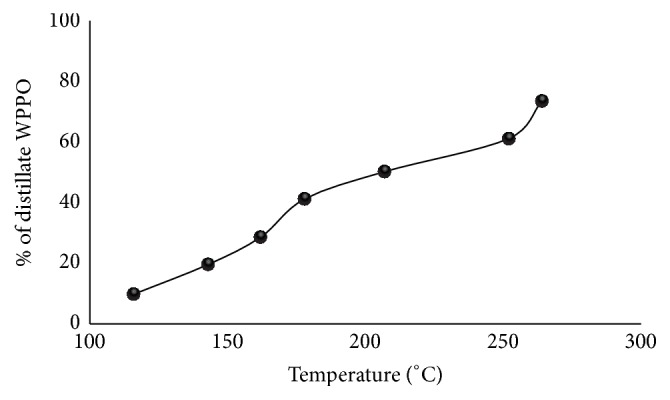
Effect of temperature on distillate product yield.

Distillation is carried out to separate the lighter and heavier fraction of hydrocarbon present in waste plastic pyrolysis oil. The distillation is operated between 116°C and 264°C; 73.5% of WPPO is distilled out. At the temperature of 116°C only about 10.0% of distilled WPPO was achieved as shown in Figure 3. However, percentage of WPPO increased constantly to 73.5% at a temperature of 264°C from 10% at the temperature 116°C.

### 3.2. Analysis of Waste Plastic Pyrolysis Oil

#### 3.2.1. Physiochemical Analysis

The waste plastic has high volatile content 77.03% by weight which is suitable for pyrolysis conversion of organic solid wastes to liquid product. The characteristics of waste plastic pyrolysis oil obtained at 425°C are shown in Table 2.

#### 3.2.2. Viscosity

**Figure 4:**
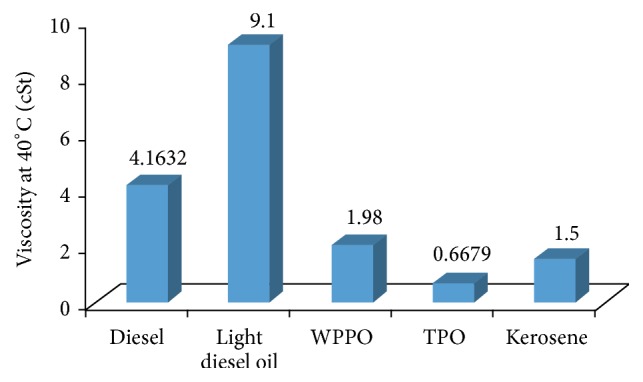
Graphical comparison of viscosity different oil. Diesel oil is 100% distillate oil, whereas light diesel oil is mixture of distillate oil and residual oil.

Viscosity varies with feedstock, pyrolysis conditions, temperature, and other variables. The higher the viscosity, the higher the fuel consumption, engine temperature, and load on the engine. On the other hand, if the viscosity of oil is too high, excessive friction may take place. The viscosity was measured by the IP-50 methodology at a temperature of 40°C. From Figure 4 it is observed that the viscosity of waste plastic pyrolysis oil obtained at 425°C pyrolysis temperature was 1.98 cSt which was comparably higher than kerosene and lower than diesel.

#### 3.2.3. Density

**Figure 5:**
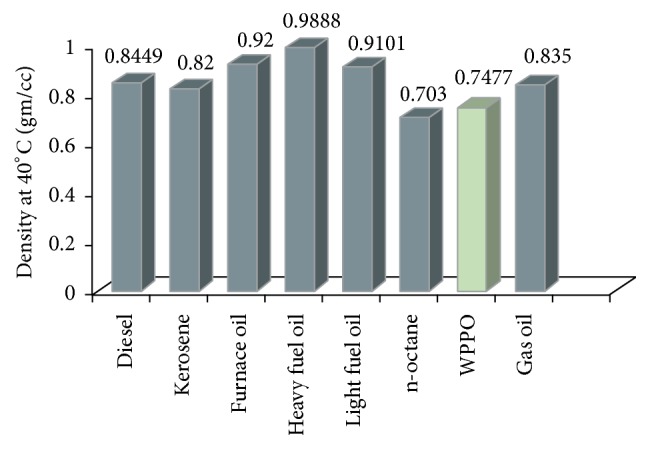
Graphical representation of density of different types of fuel.

Density is an important property of a fuel oil. If the density of fuel is high; the fuel consumption will be less. On the other hand, the oil with low density will consume more fuel which may cause damage to the engine. Therefore, too low or too high density of fuel oil is not desirable. It is clear from Figure 5 that the densities of WPPO and WPPO50 were found to be 0.7477 g/cc and 0.7943 g/cc, respectively, which is close to the density of kerosene, diesel, and gas oil. So the conventional fuel such as diesel oil, kerosene oil, and gas oil may be replaced by plastic pyrolysis oil.

#### 3.2.4. Flash Point

**Figure 6:**
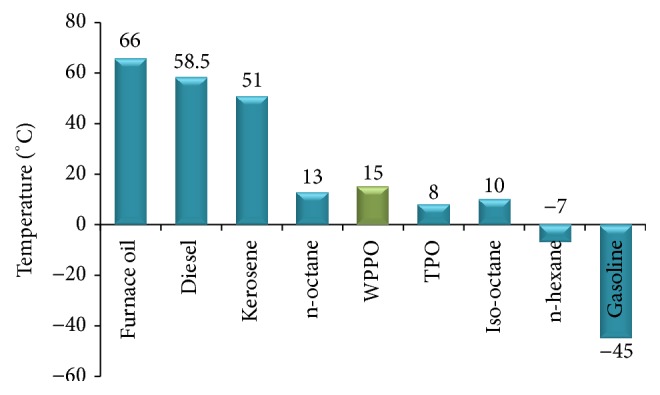
Graphical presentation of flash point of different oil.

Flash point is the lowest temperature at which it can vaporize to form an ignitable mixture in air. Flash point is used to characterize the fire hazards of fuels. The flash point of WPPO was measured according to ASTM D 93-62 method. The flash point of WPPO was about 15°C. A low flash point indicates the presence of highly volatile materials in the fuel that is a serious safety concern in handling and transporting. The flash point of furnace oil, diesel, and kerosene is higher than WPPO (Figure 6) which indicates that these are easy to handle. By removing lighter components (such as naphtha/gasoline) the flash point of WPPO will be increased.

#### 3.2.5. Fire Point and Pour Point

The fire point of a fuel is the temperature at which it will continue to burn for at least 5 seconds after ignition by an open flame. The fire point is used to assess the risk of the materials ability to support combustion. Generally, the fire point of any liquid oil is considered to be about (5–10) °C higher than the flash point. The fire point of waste plastic pyrolysis oil was 20°C.

The pour point is the temperature at which the oil will just ceases to flow when cooled at a standard rate in a standard apparatus. Pour point determines the suitability of oil for low temperature installations. The pour point of WPPO was measured by using ASTM D 97-57 methodology. The pour point was <−15°C. The low pour point value of WPPO indicates that it is not suitable in cold weather country.

#### 3.2.6. Calorific Value

**Figure 7:**
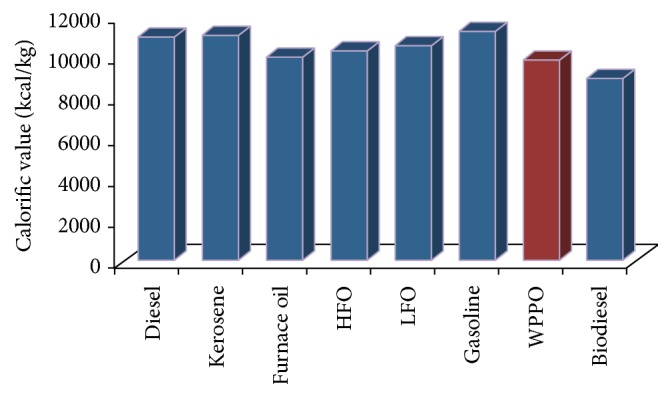
Comparison chart of calorific value of oil.

One of the important properties of a fuel on which its efficiency is judged is its calorific value. The calorific value is defined as the energy given out when unit mass of fuel is burned completely in sufficient air. The calorific value of WPPO was estimated according to IP 12/58 method. The calorific value of WPPO was 9829.3515 kcal/kg.  Figure 7 represents the comparison of calorific value of WPPO with other kinds of oil.

#### 3.2.7. Sulphur and Ash Content

The presence of sulphur in vehicle fuels causes SO_*x*_ emissions that are an environmental issue. High sulphur content decreases the catalytic conversion capacity of a system, thus increasing the emissions of nitrous oxides (NO_*x*_), carbon monoxide (CO), hydrocarbons, and volatile organic compounds (VOCs). The sulphur content of WPPO was measured by using ASTM D 129-00 methodology.

**Figure 8:**
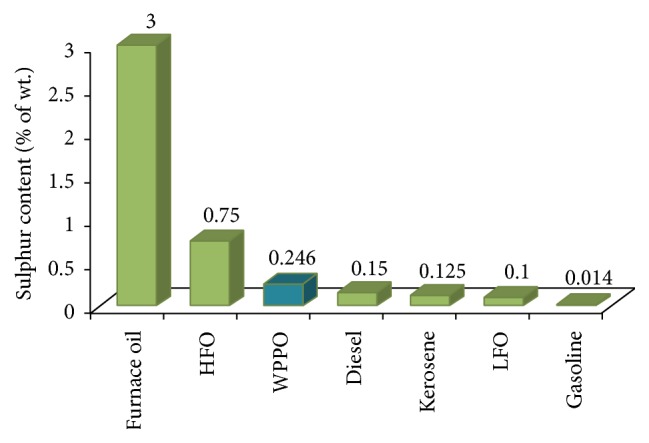
Sulphur content of different types of fuel oil.

The sulphur content of waste plastic pyrolysis oil was 0.246%. Sulphur content of WPPO is slightly higher than gasoline (0.014%), diesel (0.15%), and other types of fuel oil because waste plastic contains some contamination (Figure 8).

**Figure 9:**
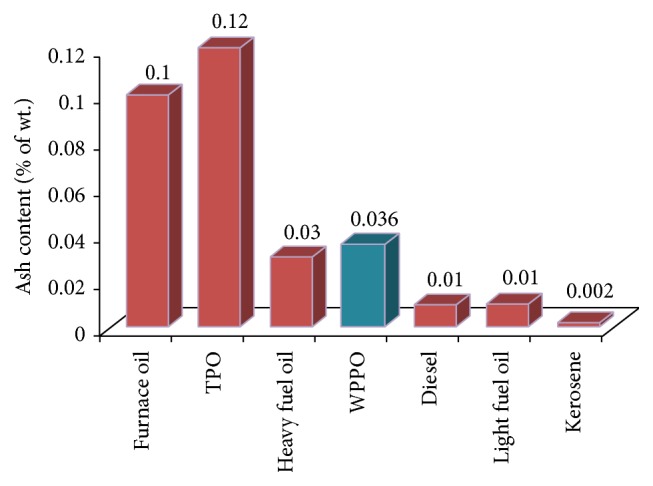
Ash content of different types of fuel oil.

The ash content of oil is the noncombustible residue. The ash content of distilled tire pyrolysis oil (DTPO) and DTPO50 (50% DTPO : 50% diesel) was measured by using IP 04/58 test methodology. From Figure 9 it is clear that the ash content of WPPO was 0.0036% comparatively higher than diesel, light fuel oil, and kerosene. So it can be used as an alternative of furnace oil and heavy fuel oil (HFO).

#### 3.2.8. Carbon Residue

Carbon residue indicates the tendency of oil to deposit a carbonaceous solid residue on a hot surface, such as a burner or injection nozzle, when its vaporizable constituents evaporate. The carbon residue of WPPO was measured according to ASTM D 189-65 method. Oil which deposits minimum amount of carbon is naturally preferable.

**Figure 10:**
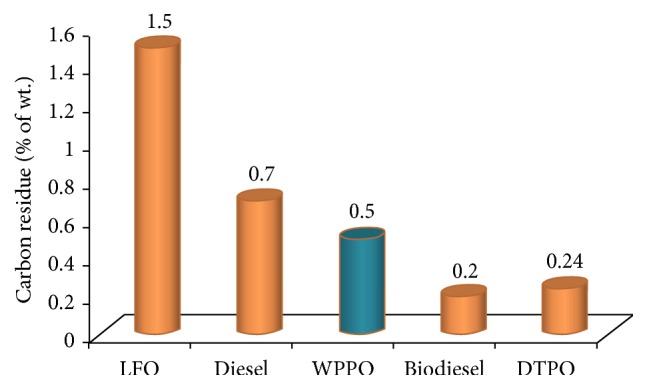
Carbon residue of different types of fuel oil.

Figure 10 shows that the carbon residue of the plastic pyrolysis oil was 0.5%. In another study, 0.05% of carbon residue was reported [22]. The carbon residue of the diesel fuel and light fuel oil was comparatively higher than WPPO. This indicates that diesel fuels will form higher deposits. Fuels with high carbon residue content could cause increased fouling of the gas ways; more frequent cleaning is necessary, especially of the turbocharger and exhaust gas boiler.

## 4. Conclusion

The thermal pyrolysis of mixed plastic leads to the production of fuel oil which is a valuable resource recovery. It also reduces the problem of disposal of waste plastic. In this work, thermal pyrolysis of waste plastic is carried out because use of catalyst is costly and regeneration of catalyst is a difficult task. Mixed plastic pyrolysis yields a mixture of oil and gas and produces very small amount of char. Higher pyrolysis temperature and longer reaction times increase the gas yield and decrease char production. Highly volatile products are obtained at low temperature. Liquid yield increases as the holding time increases from 1 hr to 2 hr, but as the holding time increases from 2 hr to 3 hr, the liquid yield decreases. The maximum oil yield was 77.03% at 2 hr. The liquid obtained in this process is relatively greater volume and low boiling range. Distillation of fuel-like liquids shows more light fractions at higher temperature and longer time. Physicochemical properties of obtained fuel oil can be exploited to make highly efficient fuel or furnace oil after blending with other petroleum products. However, further studies are necessary to utilize this oil as fuel or feedstock.

## Figures and Tables

**Table 1:** Testing methods for fuel properties measurements.

Properties	Test method
Density	IP 131/57
Kinematic viscosity	ASTM D 445
Flash point	ASTM D 93
Fire point	ASTM D 93
Water content	ASTM D 49
Pour point	ASTM D 97
Calorific value	Bomb calorimeter 12/58
Sulphur content	ASTM D 129-00
Carbon residue	ASTM D 189-65
Ash content	ASTM D 48

**Table 2:** Characteristics of waste plastic pyrolysis oil.

Properties	WPPO
Viscosity at 40°C (cSt)	1.980
Density at 40°C (g/cc)	0.7477
Carbon residue (wt%)	0.5
Ash content (%)	0.036
Sulphur content (% of wt.)	0.246
Flash point (°C)	15
Pour point (°C)	<−15
Fire point (°C)	20
Calorific value (kcal/kg)	9829.35
